# *PNPLA3* rs738409 risk genotype decouples TyG index from HOMA2-IR and intrahepatic lipid content

**DOI:** 10.1186/s12933-023-01792-w

**Published:** 2023-03-21

**Authors:** Ákos Nádasdi, Viktor Gál, Tamás Masszi, Anikó Somogyi, Gábor Firneisz

**Affiliations:** 1 Translational Medicine Institute, Faculty of Medicine, Semmelweis University, Budapest, Hungary; 2 Department of Internal Medicine and Haematology, Faculty of Medicine, Semmelweis University, Budapest, Hungary; 3 Brain Imaging Centre, Research Centre for Natural Sciences, Eötvös Loránd Research Network, Budapest, Hungary; 4 Medical Imaging Centre, Faculty of Medicine, Semmelweis University, Budapest, Hungary

**Keywords:** NAFLD, *PNPLA3*, HOMA2-IR, TyG, HTGC

## Abstract

**Background:**

Recent reports suggested a different predictive value for TyG index compared to HOMA-IR in coronary artery calcification (CAC) and other atherosclerotic outcomes, despite that both indices are proposed as surrogate markers of insulin resistance. We hypothesized a key role for liver pathology as an explanation and therefore assessed the relationship among the two indices and the intrahepatic lipid content stratified by *PNPLA3* rs738409 genotypes as a known non-alcoholic fatty liver disease (NAFLD) genetic risk.

**Methods:**

Thirty-nine women from a prior GDM-genetic study were recalled with *PNPLA3* rs738409 *CC* and *GG* genotypes for metabolic phenotyping and to assess hepatic triglyceride content (HTGC). 75 g OGTT was performed, fasting lipid, glucose, insulin levels and calculated insulin resistance indices (TyG and HOMA2-IR) were used. HTGC was measured by MR based methods. Mann–Whitney-U, χ^2^ and for the correlation analysis Spearman rank order tests were applied.

**Results:**

The *PNPLA3* rs738409 genotype had a significant effect on the direct correlation between the HOMA2-IR and TyG index: the correlation (R = 0.52, p = 0.0054) found in the *CC* group was completely abolished in those with the *GG* (NAFLD) risk genotype. In addition, the HOMA2-IR correlated with HTGC in the entire study population (R = 0.69, p < 0.0001) and also separately in both genotypes (*CC* R = 0.62, p = 0.0006, *GG*: R = 0.74, p = 0.0058). In contrast, the correlation between TyG index and HTGC was only significant in rs738409 *CC* genotype group (R = 0.42, p = 0.0284) but not in *GG* group. A similar pattern was observed in the correlation between TG and HTGC (*CC*: R = 0.41, p = 0.0335), when the components of the TyG index were separately assessed.

**Conclusions:**

*PNPLA3* rs738409 risk genotype completely decoupled the direct correlation between two surrogate markers of insulin resistance: TyG and HOMA2-IR confirming our hypothesis. The liver lipid content increased in parallel with the HOMA2-IR independent of genotype, in contrast to the TyG index where the risk genotype abolished the correlation. This phenomenon seems to be related to the nature of hepatic fat accumulation and to the different concepts establishing the two insulin resistance markers.

## Introduction

Insulin resistance (IR) refers to a decreased sensitivity of peripheral tissues to insulin and is a hallmark pathophysiologic feature of type 2 diabetes mellitus (T2DM) that clinically develops when the β-cell compensatory mechanisms might no longer overcome the increased insulin need due to the peripheral IR [1–3]. IR is closely related to the sedentary lifestyle, obesity and non-alcoholic fatty liver disease (NAFLD) that are highly prevalent [4, 5].

The most established method to assess the IR is the euglycemic–hyperinsulinemic clamp [6] developed as early as 1979, however its cost and complexity limited its everyday clinical use. There are many IR indices that are easier to assess in the clinical practice out of which HOMA-IR index appears to be the most widely used since its first description in 1985 till date [7, 8]. HOMA-IR was developed as a breakthrough model of the glucose-insulin feedback system in the homeostatic (overnight fasting) state and hepatic insulin resistance under these conditions is the major determinant of HOMA-IR [9, 10].

Nevertheless there are other surrogate markers of IR such as the triglyceride-glucose (TyG) index [11, 12] that are increasingly used due to the clinicians need to meaningfully assess IR without measuring the insulin levels. TyG index was produced in a logarithmized manner from the fasting triglycerides and glucose values and was reported to be a predictor of adverse cardiovascular outcomes in acute coronary syndrome patients with, but also without T2DM [13, 14].

Unsurprisingly both the HOMA-IR [15, 16] and the TyG index [17] are closely related to NAFLD. NAFLD is not only the most common chronic liver disease currently, but also its prevalence is doubled in patients with T2DM [4] and due to the increased IR, decreased insulin clearance [18–20] and the key metabolic functions of the liver the presence of NAFLD is a crucial factor in the speed-up of the metabolic deterioration in T2DM development and worsening of atherogenic dyslipidaemia [5, 21, 22].

Although both the HOMA-IR and TyG indices are proposed as surrogate markers of IR, recently the TyG index was found to be superior compared to the HOMA-IR in predicting coronary artery calcification (CAC) [23].

To the best of our current knowledge, the *PNPLA3* rs738409 variant shows the strongest association with NAFLD development [24]. The rs738409 gene variant and particularly the *GG* genotype is also associated with the progression of NAFLD throughout its entire spectrum [15, 25, 26], including complications occurring in patients with more advanced liver disease [27, 28].

The risk *G* allele occurs with high enough allele frequency in many of the populations worldwide, including the European (RAF: 23% [29]) and Hungarian subjects (RAF: 22%—[30]) to have a potential influence at population level.

Therefore, we have assessed the relationship between hepatic fat content, the rs738409 gene variant and the potential genotype effect on the correlation between the HOMA-IR and TyG indeces in a T2DM prone middle aged Hungarian female population.

## Patients and methods

### Participants and study design

**Fig. 1:**
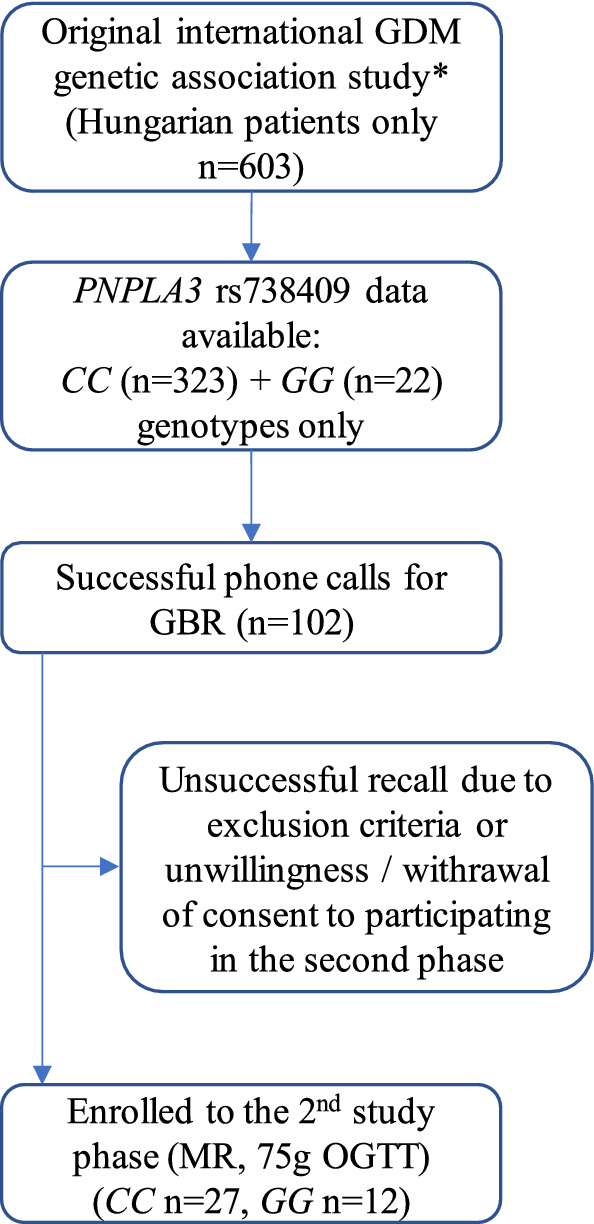
Flow chart of the genotype-based recall (GBR) study. *reference number [30]

We have access to data of over 600 Hungarian female subjects genotyped for 77 gene variants including *PNPLA3* rs738409 in a prior study [30]. A genetic based recall study (GBR) design was applied due to that GBR studies allow the assessment of genotype–phenotype associations in studies with substantially smaller samples on the basis of pre-existing genetic data resulting in a substantially higher power compared to same-sized conventional studies [31–33]. Thirty-nine women were recruited with known *PNPLA3* rs738409 genotype data: only individuals with *CC* or *GG* genotype were eligible for this study-part. A patients flow-chart is indicated on Fig. 1.

The study was conducted according to the Declaration of Helsinki after receiving approval from the relevant institutional bodies (Semmelweis University, Regional and Institutional Committee of Science, the Medical Research Council, Scientific and Research Committee of Hungary, 14486-6/2017/EKU). Participants had previously given informed consent for the whole project [30].

### Exclusion criteria

Exclusion criteria from the original case-control study [30] were extended to returning volunteers to the GBR study and included: prediabetes/T2DM diagnosed since delivery, claustrophobia and/or MR-incompatible metal implants, weight over 200 kg, waist/hip circumference ≥ 135 cm/148 cm, taking medication with a known impact on glycemic and lipid traits or HTGC, significant alcohol consumption (> 20 g/day), malignancy, or any other causes of fatty liver as detailed in the new MAFLD definition consensus statement [34]. In addition, patients with other major chronic/acute diseases or ongoing pregnancy/breastfeeding were also excluded.

### Phenotyping

Anthropometric data (age, weight, height, BMI) was recorded. 75 g OGTT was performed. Insulin and glucose levels were measured in addition to fasting „routine” laboratory parameters, including HbA1c, lipid profile and liver enzymes. From fasting trigliceride (TG), glucose and insulin levels insulin resistance indices: Trigliceride-Glucose index: TyG = ln[38,67*Tg(mg/dl)*18*glucose(mg/dl)/2], and homeostasis model assessement 2 insulin resistance index (HOMA2-IR) values were calculated [35, 36]. Diabetes mellitus (DM) and prediabetes (impaired fasting glucose (IFG)/impaired glucose tolerance (IGT)) were diagnosed according to the WHO, 2016 American Diabetes Association (ADA), and Hungarian guidelines [37–39] based on the 75 g OGTT and/or HbA1c results.

### HTGC measurement

All MR imaging (MRI) sessions were acquired on a clinical 3 T MRI system (Prisma, Siemens Healthineers, Erlangen, Germany) with the subject in a supine position. For MRI protocol, a standard body-array of 18 channel flexible coils was positioned on the liver region and combined with a spine array coil located below the subject. Imaging proton density fat-fraction (PDFF), unenhanced axial images were obtained by using a low–flip-angle, six-echo two-dimensional spoiled gradient-recalled-echo sequence with all array coil elements (TE = 2, 4.1, 6.2, 8.2, 10.2, and 12.3 ms). The repetition time and flip angle were chosen to avoid T1 weighting: TR: 15 ms, Flip-angle: 11°, FOV = 240 × 400 mm in plane, matrix = 240 × 130, slice thickness = 3.5 mm, space between slices 4.3 mm. Phase and magnitude images were systematically saved. For MR imaging using custom-made MATLAB routines, multi-section liver PDFF maps were generated offline from the source images via joint estimation of water and fat images and field maps [40]. This method uses a complex signal model similar to advanced multipoint DIXON/IDEAL algorithms (incorporating a multipeak/multifrequency fat model and T2* decay) analyzing six-echo FLASH complex images. Voxels representing liver tissue were delineated with a freehand ROI defining tool by an expert radiologist on multi-section PDFF maps excluding vessels, liver and edges, and artifacts. In each subject the mean value of the selected voxels was calculated, representing the average liver fat fraction. Fatty liver was diagnosed if the liver fat fraction exceeded 5.5% [15].

### Statistics

Due to the limited sample size and the non-normal distribution of the majority of variables we used non-parametric tests. Data are expressed in median and 25th-75th percentiles. For evaluation of the association between two parameters Spearman’s correlation test and for comparing two (genotype) groups Mann–Whitney U/χ^2^ tests were applied. TIBCO Statistica (version 13.4.0.14, TIBCO Software Inc.) software was used.

## Results

### Study population clinical characteristics

Age and BMI values of study participants were 37.0 (34–40) years and 26.2 (22.8–32.5) kg/m^2^, respectively. We diagnosed thirteen individuals with prediabetes and one with overt T2DM. No patient had established cardiovascular disease at enrolment. No significant difference of anthropometric data, glycaemic or lipid values was detected between genotype groups, except of the HTGC values (and NAFLD prevalence). The characteristic of the study population is indicated in Table 1.

**Table 1:** Population characteristic

	*PNPLA3* rs738409	
	*CC* (n = 27)	*GG* (n = 12)
Age (years)	38.0 (36.0–40.0)	35.0 (34.0–38.0)
BMI (kg/m^2^)	25.6 (21.9–32.0)	30.5 (24.8–32.8)
Overweight + obesity	15 (55%)	9 (75%)
Waist to hip ratio	0.89 (0.80–0.92)	0.92 (0.87–0.96)
GDM history	16 (59%)	6 (50%)
HbA1c %	5.5 (5.3–5.6)	5.4 (5.2–5.6)
Prediabetes^a^ + T2DM	10 (37%)	4 (33%)
HTGC (%)^b^	5.5 (2.4–5.9)^*^	11.4 (3.8–19.1)^*^
NAFLD	7 (26%)^+^	7 (58%)^+^
Fasting glucose (mmol/l)	5.2 (4.9–5.3)	5.1 (5.0–5.5)
Fasting insulin (μU/ml)	10.2 (7.2–15.9)	14.0 (9.7–19.6)
Fasting TG (mmol/l)	1.0 (0.8–1.3)	1.1 (0.8–1.5)
HOMA2-IR	1.3 (1.0–2.0)	1.8 (1.3–2.5)
TyG index	7.4 (7.3–7.8)	7.6 (7.3–7.8)

Medians (25th-75th percentile) or Number of patients (%) are indicated

^a^Diagnosed by using HbA1c and/or glucose values from OGTT (IFG, IGT)

^b^Six women had an indeterminate (− 1.455–0.676) NFS value, of which 4 patients had prediabetes/T2DM and none had pathologic FIB-4 score (> 1.45) or routine abdominal MRI scans indicating advanced liver fibrosis/cirrhosis

GDM: gestational diabetes mellitus, T2DM: type 2 diabetes mellitus, HTGC: hepatic triacylglycerol content, NAFLD: non-alcoholic fatty liver disease, NFS: NAFLD fibrosis score, FIB-4 score: fibrosis-4 score

^*^p = 0.01,^+^p = 0.05

### Correlation between TyG and HOMA2-IR indices stratified by *PNPLA3* rs738409 genotype

**Fig. 2:**
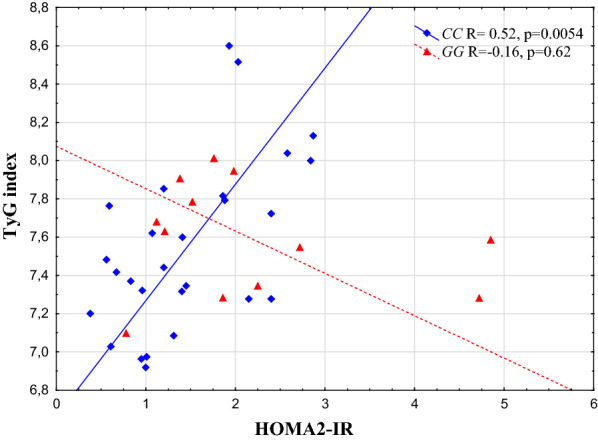
Correlation between HOMA2-IR and TyG indices stratified by *PNPLA3* rs738409 genotypes. Spearman rank order test results indicated in the figure

Between the two insulin resistance indices we found an association (R = 0.42, p = 0.0078), however a significant correlation was only detectable in individuals with the rs738409 *CC* genotype (R = 0.52, p = 0.0054) but not with *GG* (r = − 0.16, p = 0.62). The scatterplot is indicated in Fig. 2.

### Genotype effect on correlations among insulin resistance indices, their components and HTGC

HTGC values significantly correlated with both markers of insulin resistance: (TyG R = 0.36, p = 0.0252, HOMA2-IR: R = 0.69, p < 0.0001), and their components (fasting glucose: R = 0.51, p = 0.0008, fasting insulin: R = 0.66, p < 0.0001, fasting TG: R = 0.32, p = 0.0445).

**Fig. 3:**
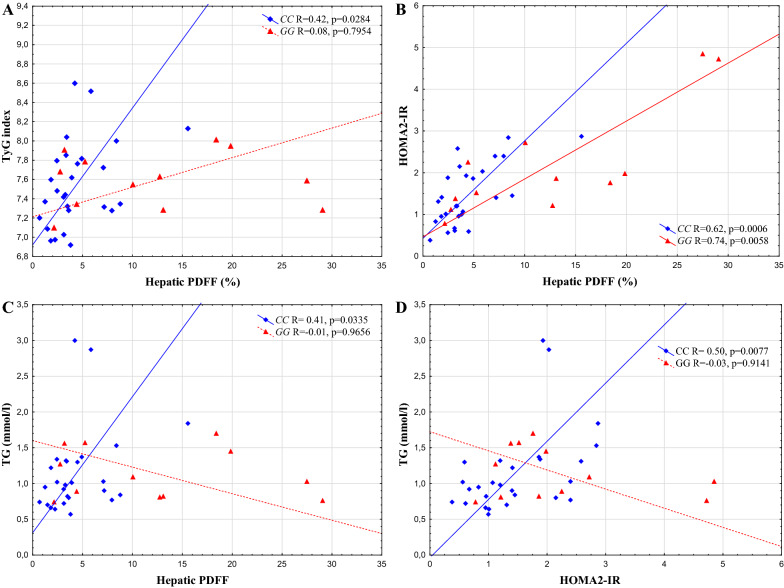
Correlations among HTGC, insulin resistance indices and TG values stratified by *PNPLA3* rs738409 genotypes (*CC* vs *GG*): **A**: between HTGC and TyG index, **B**: between HTGC and HOMA2-IR, **C**: between HTGC and TG, **D**: between HOMA2-IR and TG. Spearman rank order test results indicated in the figure. HTGC: hepatic triacylglycerol content, TG: fasting serum triglyceride

After genotype stratification In contrast to HOMA2-IR and its components, where the correlations were nearly parallel in the two genotype groups after genotype stratification, the correlations of TyG index and fasting TG were only significant in the rs738409 CC genotype group indicating different associations between the parameters according to the genotypes to HOMA2-IR and its components, where the correlations were nearly parallel in the two genotype groups, the correlations of TyG index and fasting TG were only significant in the rs738409 *CC* genotype group indicating different associations between the parameters according to the genotypes. Similarly, the correlation between HOMA2-IR and fasting TG was disrupted in individuals with *GG* genotype. Correlations between HTGC and TyG, HTGC and HOMA2-IR, HTGC and TG, HOMA2-IR and TG are indicated in Fig. 3A, B, C, D, respectively.

## Discussion

We assessed intrahepatic lipid content measured by MR method and two indices proposed as surrogate markers of insulin resistance indices (TyG and HOMA2-IR) in 39 middle-aged women prone to T2DM development. Genotype based recall study design was applied on the basis of *PNPLA3* NAFLD risk genotype to decrease the number of patients needed to be enrolled [31, 33]. Therefore, only patients with rs738409 homozygous genotypes (*GG* vs *CC*) were included. Despite the two IR indices were suggested to have a linear relationship [17, 41], we found compelling difference in the relationship between TyG and HOMA2-IR when the data were stratified by the *PNPLA3* genotypes. The profound genetic effect on the correlations between TyG, TG and HTGC and also between TG and HOMA2-IR was observed.

Insulin resistance and hyperinsulinemia are classified as major factors contributing to pathology of T2DM development and its cardiovascular complications. Many concepts and methods were developed to measure the “insulin resistance” (IR), however no single parameter could meet the need of a precise pathophysiological assessment combined with an easy use for the everyday clinics. Out of the many IR indices HOMA-IR index appears to be the most widely used in the clinical practice, including the recent recommendation to establish the diagnosis of metabolic (dysfunction) associated fatty liver disease (MAFLD) [34]. The HOMA-IR and the further developed HOMA2-IR are proposed to be mainly associated with hepatic insulin resistance under fasting steady-state conditions [8, 36, 42]. Despite HOMA-IR is easily calculated from only two parameters there is a need from clinicians to find alternative surrogate markers of IR which do not require the measurement of fasting insulin levels such as the triglyceride-glucose (TyG) index [12].

Two markers of insulin resistance, the HOMA-IR and TyG were compared in a recent study on the prognostic value of T2DM development and atherosclerosis and the authors concluded that TyG index performed better in both endpoints [43].

Nevertheless there is still no clear-cut opinion in the literature regarding the predictive powers of the TyG and HOMA-IR index in CVD, and studies suggested only a moderate coronary artery disease (CAD) predictive power for TyG, although its inclusion into predictive models of MACEs could improve the predictive accuracy in patients with ACS [44].

Due to that hepatic insulin resistance is significantly increased in NAFLD that is the most prevalent liver disease and strongly related to obesity, T2DM, dyslipidaemia and metabolic syndrome, the pathophysiology of the liver lipid accumulation could be a key factor in (hepatic) IR associated to the common metabolic diseases. Therefore we assessed the effect of *PNPLA3* rs738409 gene variant on the correlation between TyG and HOMA2-IR. This gene variant was identified in fatty liver genome-wide association studies (GWAS) [24] with pathogenic role throughout the entire NAFLD spectrum [25, 27, 28].

We astonishingly found that TyG and HOMA2-IR indices were completely dissociated in individuals with the rs738409 *GG* risk genotype (M148M) in contrast to those with the *CC* (I148I) genotype where the strong positive correlation was conserved.

This finding should mean that despite both the TyG and the HOMA2-IR are considered as surrogate markers of insulin resistance they display fundamentally different characteristics according to the genetic background of the liver lipid accumulation confirming the hypothesis of the *PNPLA3* rs738409 genotype effect.

Although prior studies with human liver tissue samples reported that the hepatic content of diacylglycerol (DAG) species (FA18:1, implicated in diminished insulin signaling) remained unaltered in rs738409 homozygous and heterozygous *G* allele carriers (M148M and I148M) and it was hypothetically suggested that the *G* risk allele carriers might be protected from insulin resistance [45] we report here that the HOMA2-IR values remained correlated with HTGC. In addition, our observation is consistent is consistent with prior results where HOMA-IR/HOMA2-IR and HTGC values were directly measured and reported to be correlated in subjects with *PNPLA3* risk genotypes [16]. In contrast to the HOMA2-IR only the TyG index displayed the theoretically expected characteristic in correlation with HTGC in individuals homozygous for the *PNPLA3* NAFLD risk genotype based on the reported hepatic DAG species content critical in disturbing the insulin signal [45].

We observed that the correlation between TyG and HTGC was diminished in those with the rs738409 *GG risk* genotype. The explanation could be attributed to that the input parameters and their *PNPLA3* risk genotype associated changes are largely different. The insulin concentrations were increasing in parallel with HTGC, in contrast to the glucose levels that were not different. Consistently, the fasting insulin levels and the HOMA-IR indices were significantly higher and the glucose levels were unchanged in a severely obese large European cohort in the rs738409 *GG* genotype group [46] that was presumably presented with higher HTGC values as well. Opposingly, the serum TG levels in the morbid obese patients with the *GG* genotype was found to be lower compared to *CC* genotype group [46] and confirmed subsequently [47, 48]. The lower circulating TG levels in the *PNPLA3* risk genotype group could be due to the lower hepatic TG efflux in the fasting state associated to the genetic effect on intrahepatocellular lipolysis [46, 48, 49]. Based on that the hepatic VLDL output occurs in parallel with the TG efflux in the fasting state [50] these observations might well explain that the TyG index could be an useful prognostic marker for CAD, especially in high CVD risk patients with T2DM [43, 44, 51–53]. Furthermore, in a recent study with over 4000 participants undergoing cardiac CT the TyG index was found to be superior compared to the HOMA-IR in predicting CAC [23]. It could be outlined that the rs738409 *G* allele frequencies in the East Asian populations are substantially higher compared to populations with European or African origin [29] which is consistent with the results of the latter East Asian study [23] and could contribute to the difference found between TyG index and HOMA-IR in predicting CAC.

The current state of the art is that HOMA-IR primarily reflects hepatic insulin resistance [10]. The increase of HOMA2-IR occurred in parallel with the increase of HTGC in those with the *PNPLA3* rs738409 *GG* risk genotype and partially behaves like a biomarker of intrahepatic steatosis that is dissociated from the other surrogate marker of IR, the TyG index composed of only metabolic parameters but does not directly include serum insulin level.

As a consequence, it may also be raised, that HOMA-IR could be significantly dissociated from the TyG index in those populations where the rs738409* G* NAFLD risk allele is the major *PNPLA3* allele [29, 54].

The authors suggest that the genetic background of the pathologic liver lipid accumulation and its relation to HOMA-IR and TyG index are important insights towards a better understanding, more precise interpretation and perhaps towards a better clinical use of these IR estimates.

### Limitations

Our results were limited by the sample size and the lack of supplementary methods to study the underlying pathology. Further studies are needed to replicate our findings and to confirm our results in other populations.

## Data Availability

The datasets used and analysed during the current study are available from the corresponding author on reasonable request.

## Abbreviations

BMI: Body mass index
FIB-4: Fibrosis 4 score
GBR: Genetic-based recall (study)
GDM: Gestational diabetes mellitus
HOMA: Homeostasis model assessment
IFG: Impaired fasting glucose
HTGC: Hepatic triglyceride content
IGT: Impaired glucose tolerance
IR: Insulin resistance
MR: Magnetic resonance
NAFLD: Non-alcoholic fatty liver disease
NFS: NAFLD fibrosis score
OGTT: Oral glucose tolerance test
PDFF: Proton density fat-fraction
*PNPLA3*: *Patatin-like phospholipase domain-containing protein 3*
TyG index: Triglyceride-glucose index
T2DM: Type 2 diabetes mellitus
TG: Triacylglycerol
W/H: Waist-to-hip ratio
